# Changes of the hypothalamic-pituitary-thyroid axis in aging

**DOI:** 10.1186/1756-6614-6-S2-A49

**Published:** 2013-04-05

**Authors:** Monika Puzianowska-Kuźnicka

**Affiliations:** 1 Department of Geriatrics and Gerontology, CMKP, Warsaw, Poland; 2 Department of Human Epigenetics, IMDiK PAN, Warsaw, Poland

## 

Study of healthy aging of the hypothalamic-pituitary-thyroid axis is difficult, since aging is accompanied by the increased frequency of various thyroid diseases and of extrathyroidal disease, as well as by the increased intake of medicines modulating the function of this axis. Nevertheless, researchers managed to determine that in the paraventricular nucleus of aged humans the number of neurons synthesizing TRH is increased. In the pituitary, the number of thyrotrophs also increases, and the cells are commonly hypertrophic. In the thyroid, epithelial cells are smaller, the amount of colloid decreases, and the whole gland becomes smaller and denser. It is still not known how aging affects secretion of TRH. Healthy aging is accompanied by the slight increase of concentration of TSH (highest concentrations of this hormone were detected in centenarians), and the night peak of its secretion is lower. In healthily aging humans, concentrations of T3 and of fT3 decrease, concentration of rT3 increases, and concentrations of T4 and of fT4 remain stable.

According to the current theory of aging, the direct cause of aging is the accumulation of stochastic damage to DNA, and to proteins and lipids. The most important (albeit not only) damaging factors are the reactive oxygen species (ROS), by-products of the cellular metabolism, especially of the respiratory chain in mitochondria. Most likely, the function of the hypothalamic-pituitary-thyroid axis influences the rate of aging, since the function of the respiratory chain and, therefore, the rate of production of ROS, remain under the control of T3. Consequently, slowdown of the T3-dependent metabolism might promote longer life through slower production of ROS. This hypothesis is supported by the above described epidemiological observations showing that healthy aging is accompanied by the decreased activity of the hypothalamic-pituitary-thyroid axis and by the fact that in long-lived humans (whole 85+ group and, especially, centenarians) the activity of this axis is lowest compared to younger age groups. Because of this, subclinical hypothyroidism in 65 years old or older humans might be, in fact, a manifestation of healthy aging of the thyroid gland, and this is why in these individuals this condition should not be “automatically” treated. Instead, the patient should be regularly examined (if we do not want to omit the overt hypothyroidism), and decision to treat should be taken individually, based on the biological age, the clinical state, the presence of other diseases, the results of biochemical tests, etc.

The percentage of aged humans with anti-TPO and anti-TG auto-antibodies is much higher compared to young individuals, but above the age of 80 the percentage of such individuals decreases. Aging is accompanied by the increased frequency of hypothyroidism, hyperthyroidism, and of thyroid cancers. Their symptoms are commonly untypical and, because of this, might be confused with signs of aging per se or with symptoms of other diseases. Nevertheless, all these diseases should be adequately diagnosed and treated.

